# SVExpress: identifying gene features altered recurrently in expression with nearby structural variant breakpoints

**DOI:** 10.1186/s12859-021-04072-0

**Published:** 2021-03-21

**Authors:** Yiqun Zhang, Fengju Chen, Chad J. Creighton

**Affiliations:** 1 Dan L. Duncan Comprehensive Cancer Center Division of Biostatistics, Baylor College of Medicine, Houston, TX 77030 USA; 2 Department of Bioinformatics and Computational Biology, The University of Texas MD Anderson Cancer Center, Houston, TX 77030 USA; 3 Human Genome Sequencing Center, Baylor College of Medicine, Houston, TX 77030 USA; 4 Department of Medicine, Baylor College of Medicine, Houston, TX 77030 USA

**Keywords:** Cancer, Structural variation, Genomic rearrangement, Whole genome sequencing, CCLE, Data integration, Multiplatform analysis

## Abstract

**Background:**

Combined whole-genome sequencing (WGS) and RNA sequencing of cancers offer the opportunity to identify genes with altered expression due to genomic rearrangements. Somatic structural variants (SVs), as identified by WGS, can involve altered gene *cis*-regulation, gene fusions, copy number alterations, or gene disruption. The absence of computational tools to streamline integrative analysis steps may represent a barrier in identifying genes recurrently altered by genomic rearrangement.

**Results:**

Here, we introduce SVExpress, a set of tools for carrying out integrative analysis of SV and gene expression data. SVExpress enables systematic cataloging of genes that consistently show increased or decreased expression in conjunction with the presence of nearby SV breakpoints. SVExpress can evaluate breakpoints in proximity to genes for potential enhancer translocation events or disruption of topologically associated domains, two mechanisms by which SVs may deregulate genes. The output from any commonly used SV calling algorithm may be easily adapted for use with SVExpress. SVExpress can readily analyze genomic datasets involving hundreds of cancer sample profiles. Here, we used SVExpress to analyze SV and expression data across 327 cancer cell lines with combined SV and expression data in the Cancer Cell Line Encyclopedia (CCLE). In the CCLE dataset, hundreds of genes showed altered gene expression in relation to nearby SV breakpoints. Altered genes involved TAD disruption, enhancer hijacking, and gene fusions. When comparing the top set of SV-altered genes from cancer cell lines with the top SV-altered genes previously reported for human tumors from The Cancer Genome Atlas and the Pan-Cancer Analysis of Whole Genomes datasets, a significant number of genes overlapped in the same direction for both cell lines and tumors, while some genes were significant for cell lines but not for human tumors and vice versa.

**Conclusion:**

Our SVExpress tools allow computational biologists with a working knowledge of R to integrate gene expression with SV breakpoint data to identify recurrently altered genes. SVExpress is freely available for academic or commercial use at https://github.com/chadcreighton/SVExpress. SVExpress is implemented as a set of Excel macros and R code. All source code (R and Visual Basic for Applications) is available.

**Supplementary Information:**

The online version contains supplementary material available at 10.1186/s12859-021-04072-0.

## Background

In cancer, somatic structural variations (SVs) are rearrangements of large DNA segments within the cancer genome. SVs may impact nearby genes’ expression in several ways, including forming fusion transcripts or disrupting or repositioning cis-regulatory elements near genes. Our recent studies [1–4] have demonstrated an analysis approach to integrate SV with gene expression data, identifying gene-level associations between altered expression and nearby SV breakpoints in proximity to genes. Genes recurrently deregulated in conjunction with SVs may involve topologically associated domain (TAD) disruption or enhancer hijacking. An SV involves two breakpoints representing the fusion of two respective genomic coordinates. Some of the individual steps involved in our SV-expression integrative analysis approach include constructing a gene-to-sample breakpoint pattern matrix, linear regression modeling to associate altered expression with nearby breakpoints, and identifying putative enhancer hijacking and TAD disruption events. These steps would be labor-intensive for most analysts working from scratch using standard tools such as R, Excel, or BEDtools [5]. The absence of computational tools to streamline these steps may represent a barrier to others' ability to implement our approach in other datasets.

Other published methods for integrating SV with expression data include cis-X [6], which analyzes data from a single cancer sample. cis-X first finds aberrantly cis-activated genes that exhibit allele-specific expression accompanied by an elevated outlier expression, then searches for causal noncoding variants including SV-associated enhancer hijacking. In contrast, our data integration method utilizes large sample cohorts, rather than a single sample, to identify genes recurrently impacted by SVs across multiple samples. Another software package, SV-HotSpot [7], identifies hotspots of SV breakpoints represented by a set of cancer samples, which hotspots may then be evaluated for expression associations involving nearby genes. In contrast, our method does not focus exclusively on hotspot patterns, as we have found that SVs contributing to deregulated expression may involve breakpoints across a large region surrounding a given gene, not limited to hotspots nor to a single mechanism [1–4]. Our method is similar in many respects to the Cis Expression Structural Alteration Mapping (CESAM) method [8, 9], which also relies on linear regression modeling to integrate expression with SV breakpoint pattern across a large number of samples. However, for the gene-to-sample breakpoint matrix, CESAM assigns SV breakpoints to bins if they fall into the same pre-annotated TAD. In contrast, our method does not limit itself to TAD disrupting SVs or potential enhancer hijacking events. No public software tool for using the CESAM method appears to be available. Whereas the linear modeling steps involved with our method or CESAM should be relatively straightforward for bioinformatics users to carry out using R, no user-friendly software has been available for compiling SV data into a form amenable for linear modeling with expression data.

As presented here, our “SVExpress” suite of computational tools allows one to identify SV breakpoint-to-expression associations across a set of cancer samples profiled for both SVs and gene transcription. SVExpress takes as input a table of somatic SV breakpoints, a gene-to-sample expression matrix, and a gene-to-sample copy number alteration (CNA) matrix. Using Excel Visual Basic for Applications (VBA), SVExpress then constructs a gene-to-sample breakpoint matrix, which the user can then integrate with the expression matrix by linear regression modeling, using the provided R code. Furthermore, using SVExpress, top SV-gene associations identified can be examined in terms of enhancer hijacking (e.g., the positioning of an enhancer represented by one breakpoint in proximity to a gene nearby the other breakpoint) or in terms of disruption of TADs. Genomic datasets involving hundreds of cancer sample profiles can be readily analyzed using our tools. SVExpress is intended to be usable for those who may not necessarily have programming or computational skills, as well as bioinformaticians. As a demonstration of SVExpress, here, we also analyze SV and expression data across 327 cancer cell lines in the Cancer Cell Line Encyclopedia (CCLE) [10].

## Implementation

### Generating a gene-to-sample SV breakpoint matrix

**Fig. 1:**
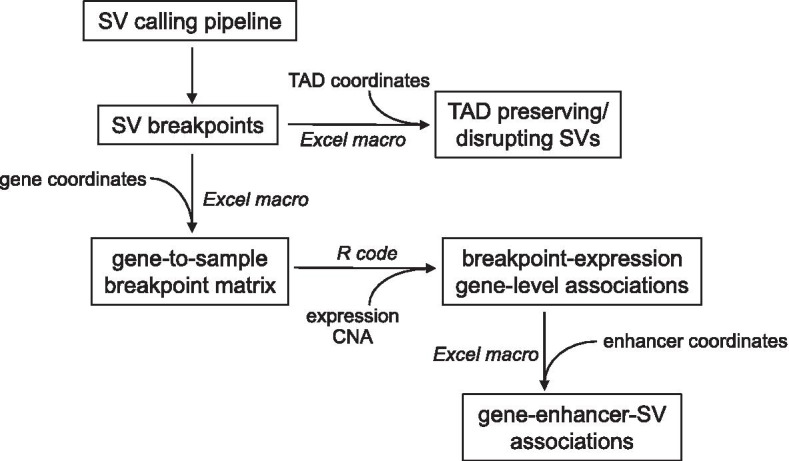
Workflow diagram for the SVExpress suite of computational tools. SVExpress identifies SV breakpoint-to-expression associations across a set of cancer samples profiled for both SVs and gene transcription. Initially, SVExpress takes as input a table of SV breakpoints (which may be generated using any standard SV calling algorithm) and a set of gene coordinates. SVExpress then constructs a gene-to-sample breakpoint matrix using an Excel macro (“Generate_Gene_to_Sample_SV_Table”). The user can then take this breakpoint matrix and integrate it with the corresponding matrices for gene expression and gene-level copy number alteration (CNA) by linear regression modeling using the provided R code. This code generates *p* values and t-statistics for each gene, associating SV breakpoint pattern with expression, with or without correcting for CNA. Furthermore, using SVExpress Excel macros, a set of SV-gene associations identified can be examined in terms of enhancer hijacking (e.g., an enhancer represented by one breakpoint positioned in proximity to a gene nearby the other breakpoint) or in terms of disruption of TADs. SVExpress carries out the above using the "Generate_SV_to_Enhancer_Associations" and "Generate_SV_to_TAD_Associations" macros, respectively. SV, Structural Variant; CNA, copy number alteration; TAD, topologically associated domain

Figure 1 provides a workflow diagram for the SVExpress suite of computational tools. In Excel, the user assembles a workbook with a table of somatic SV breakpoints (using the standard output from any of the commonly used SV calling algorithms) and another table of the coordinates for all genes. An Excel macro then generates a gene-to-sample SV breakpoint matrix, based on the user-specified region of interest relative to each gene. For example, breakpoints occurring within a given gene could involve gene fusions or disruption of tumor suppressor genes [3], and breakpoints falling within a larger region (e.g., ~ 1 Mb) surrounding the gene could involve enhancer hijacking events [2, 3]. Using a 1 Mb region surrounding each gene, the user may specify the “relative distance metric” option [2], whereby breakpoints that occur close to the gene will have more numeric weight in identifying SV-expression associations, while breakpoints further away but within 1 Mb can have some influence. When not using the 1 Mb distance metric option, the gene-to-sample matrix entries are 1, if a breakpoint occurs in the specified region for the given gene in the given sample, and 0 if otherwise. The generated breakpoint matrix is for the linear modeling step involving the SVExpress R code (see below). The macro can also generate the identifiers related to the set of gene-to-SV associations used to construct the matrix (using the SV breakpoint closest to the gene start if multiple breakpoints are in the given region). These gene-to-SV associations can then be examined for putative enhancer hijacking events or TAD disruption (see below).

### Generating gene-to-breakpoint expression correlations

The user assembles gene-to-sample data matrices for expression, CNA, and SV breakpoint pattern (the latter matrix assembled using the above SVExpress Excel macro). Then, R code provided as part of SVExpress carries out linear modeling to assess for each gene the correlation between its expression and the presence of nearby SV breakpoints. Multiple linear models for each gene may be considered, including models that correct for gene-level CNA. As genomic rearrangements are often associated with widespread CNA patterns [1–4], SV-expression associations that would remain significant in models incorporating CNA would likely be of primary interest. For each model considered (with or without CNA as a covariate), results provide the t-statistic and *p *value for each gene's expression versus breakpoints correlation. Given the significance *p *values for all genes, corrections for multiple testing can use standard methods such as Storey and Tibshirani [11]. If any technical batch effects are present in the expression or CNA data, these should be corrected before the linear modeling step (e.g., using Combat [12]).

### Associating enhancers with gene-to-SV mappings

SVExpress can search a given set of gene-to-breakpoint associations (defined using the first SV breakpoint) for potential enhancer translocation events represented by the second SV breakpoint. Using the provided Excel macros and an input table of SV breakpoints, SVExpress can generate a set of gene-to-SV associations involving each sample. For each association, SVExpress examines the region 1 Mb from the other SV breakpoint for any enhancers repositioned upstream in proximity to the gene. SVExpress also identifies any enhancers located within 1 Mb upstream of the unaltered gene. The user may then wish to compare the number of enhancer hijacking events found for the subset of gene-to-sample-to-SV associations involving gene over-expression with the number of enhancer hijacking events found for the entire set of gene-to-sample-to-SV associations. Previously, we have observed a significant enrichment of putative enhancer hijacking events involved with the set of SVs associated with gene over-expression [1–4]. For the above, the user assembles a table of enhancer coordinates, which may come from several data sources, including the ENCODE project [13].

### Associating TADs with SVs

An SVExpress macro searches a given set of SV breakpoints and note whether each SV is “TAD preserving” or “TAD disrupting.” For TAD preserving SVs, both SV breakpoints locate within the same TAD. For TAD disrupting SVs, the SV breakpoints span the boundaries of different TADs. For SVs associated with gene over-expression, we have previously observed a significant enrichment of TAD-disrupting SVs [2, 3]. The user assembles a table of TAD boundaries for the above, which may come from other studies [14].

## Results

### Global impact of SVs on gene expression patterns in cancer cell lines

As a demonstration here of SVExpress, we used it to assess gene-level associations between expression and nearby somatic SV breakpoints across 327 cancer cell lines in the Cancer Cell Line Encyclopedia (CCLE) with Whole Genome Sequencing (WGS) data. The CCLE datasets were from the 2019 release [10], with gene-level RSEM expression calls by RNA sequencing (RNA-seq). Somatic SV calls were previously made in these cell lines by SVABA algorithm [10]. For each gene with expression data, we assessed the pattern of nearby SV breakpoints within each of a set of region windows: 100 kb upstream of the gene, 100 kb downstream of the gene, within the gene body, and 1 Mb upstream or downstream of the gene. Using the SVExpress Excel macro, we assembled a data matrix of breakpoint patterns for 20,153 unique named genes and 327 cell lines. Using the SVExpress R code, we then assessed the association between expression and SV breakpoint pattern for each gene by linear models correcting for cancer type and gene-level CNA.

**Fig. 2:**
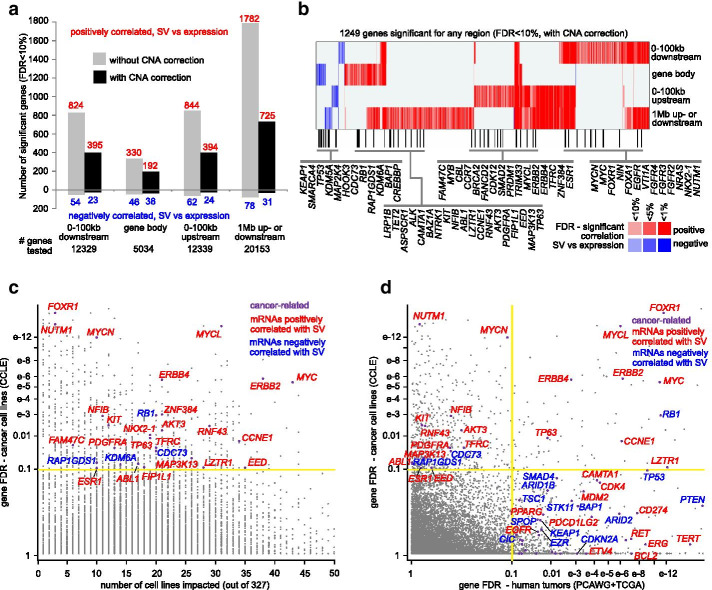
Genes with altered expression associated with nearby SV breakpoints across 327 cancer cell lines. **a** For each of the indicated genomic region windows examined, numbers of significant genes (FDR < 10%) showing a correlation between expression and associated SV event across 327 cancer cell lines with WGS and expression data [10]. Numbers above and below the zero point of the y-axis denote positively and negatively correlated genes, respectively. Linear regression models evaluated significant associations when correcting for cancer type (gray) and for both cancer type and gene-level CNA (black). For the 1 Mb region window, the model weights the relative gene distances of the breakpoints [2]. **b** Heat map of significance patterns for 1249 genes significant for any region window (FDR < 10%, correcting for both cancer type and CNA). Red denotes significant positive correlation; blue, significant negative correlation. Genes listed are cancer-associated [23]. **c** Significance of genes in cancer cell lines, as plotted (Y-axis) versus the number of cell lines impacted (expression > 0.4SD from sample median) by nearby SV breakpoint (within 1 Mb). **d** Significance of genes in combined PCAWG-TCGA cohort (2334 patients, x-axis) [2], as compared to their significance in the cancer cell line cohort (327 cell lines, y-axis) [10]. Genes in the upper left quadrant reached significance only in the 327-cancer cell line dataset. For parts c and d, significant genes are defined by 1 Mb region window, correcting for tumor type and CNA, and “cancer-related” is by COSMIC [23]. SV, Structural Variant; FDR, False Discovery Rate; CCLE, Cancer Cell Line Encyclopedia; PCAWG, Pan-Cancer Analysis of Whole Genomes; TCGA, The Cancer Genome Atlas

Hundreds of genes showed altered gene expression in relation to nearby SV breakpoints, including breakpoints located either downstream or upstream of genes and breakpoints occurring in the gene body (Fig. 2a and Additional file 1: Data File S1). Incorporating statistical corrections for gene-level CNA decreased the overall numbers of significant genes, reflecting previous observations of global associations of SV breakpoints with copy number gain [1, 3]. Many more genes showed positive correlations with SV breakpoints (i.e., expression tended to be higher when a nearby SV breakpoint was present) than negative correlations. When considering a 1 Mb region window upstream or downstream of each gene (using our previous described “distance metric” model [2], with corrections for tumor type and CNA), 725 genes showed positive correlations with SV breakpoints, independent of CNA, and 31 genes showed negative correlations (FDR < 10%[11]). Genes positively correlated with SV breakpoints included many known oncogenes, while genes negatively correlated included many known tumor suppressor genes (Fig. 2b). Within-gene SV breakpoints may disrupt tumor suppressors [3], as observed here for such genes as *TP53*, *RB1*, and *KEAP1*.

We had previously analyzed WGS and expression data from the combined PCAWG-TCGA cohort of human tumors across many tissue types (2334 patients), to define the set of genes recurrently altered in association with SV breakpoints [2]. When comparing results from CCLE cell lines with results from human tumors, a significant number of genes overlapped in the same direction, while some genes were significant in one dataset but not the other (Fig. 2c, d). Focusing on the 1 Mb region surrounding each gene (and correcting for cancer type and CNA), 82 genes with FDR < 10% for each dataset overlapped between both results sets, a highly significant overlap (*p* < 1E−14, one-sided Fisher’s exact test, Fig. 2d). Oncogenes that were significant for CCLE cell lines but not for PCAWG-TCGA human tumors included *MYCN*, *NUTM1*, and *ESR1*. Genes significant for human tumors but not for cell lines included oncogenes *TERT*, *BCL2*, *RET*, *ERG*, *MDM2*, *CDK4*, and tumor suppressor genes *PTEN*, *STK11*, and *CDKN2A*. However, some of the above genes not significant using the 1 Mb region in the CCLE dataset were significant for one of the other regions examined (Additional file 1: Data File S1). Differences between the two sets of results likely originate in part from the different cancers represented in the respective datasets.

### SV-associated TAD disruption and enhancer hijacking events in cancer cell lines

**Fig. 3:**
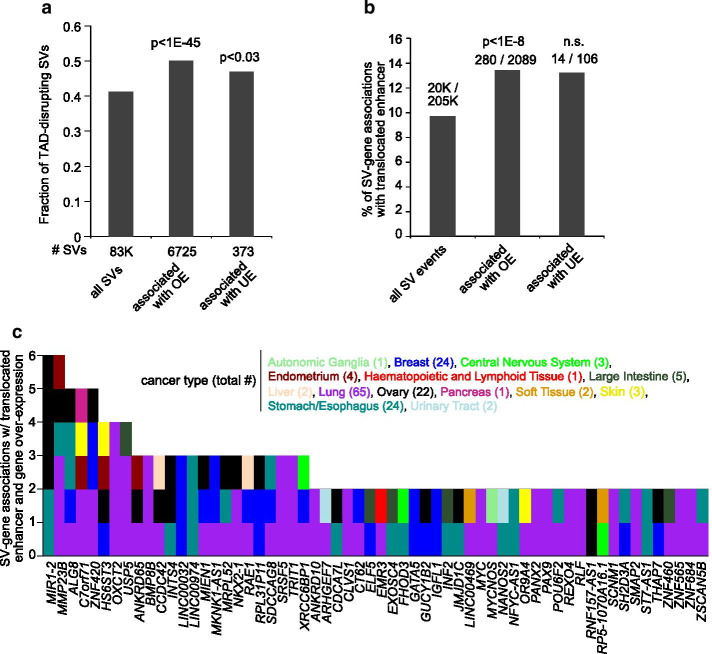
SVs associated with disruption of TADs and translocated enhancers in cancer cell lines. **a** As compared to all SVs, fractions of SVs involving topologically associated domain (TAD) disruption and altered gene expression (defined as FDR < 10% for the gene using 1 Mb region window, with corrections for tumor type and CNA, and expression > 0.4SD or ≤ 4SD from the median for the case harboring the breakpoint). Results based on analysis of 327 cancer cell lines with WGS data [10]. *p* values by chi-squared test. **b** Percentages of SV breakpoint associations involving the translocation of an enhancer within 0.5 Mb of the SV breakpoint in proximity to the gene (and closer than any enhancer within 1 Mb of the unaltered gene), as tabulated for the entire set of SV breakpoint associations with breakpoint mate on the distal side from the gene, as well as for the subsets of SV breakpoint associations involving altered gene expression (defined as for part a). *P* values by chi-squared test. **c** By gene and by cancer type, the number of SV breakpoint associations involving the translocation of an enhancer, involving at least two cell lines per gene. Results involve 60 genes and 159 cell lines

Using SVExpress macros, we could assess the fraction of positive SV-expression correlations that seem to reflect SV-mediated disruption of TADs or enhancer hijacking. From input data on TAD coordinates in human cells [13, 14], an SVExpress macro categorized all SVs in the CCLE dataset by those that were TAD disrupting versus those that were non-disrupting. The subset of SVs involving genes over-expressed in conjunction with SV breakpoints were significantly enriched for TAD-disrupting SVs (Fig. 3a *p* < 1E−45, chi-squared test), consistent with previous observations in human tumors [3]. Using other SVExpress macros, we generated all SV breakpoints-to-gene associations occurring within 1 Mb of each other. We then examined the translocated region represented by the SV breakpoint upstream of each gene for any involved enhancers [13]. SV breakpoints associated with over-expressed genes were significantly enriched (*p* < 1E−8, chi-squared test) for putative enhancer translocation events, with the rearrangement bringing an enhancer within 500 kb of the gene (Fig. 3b), involving 181 over-expressed genes and 145 cell lines (Fig. 3c and Additional file 1: Data File S1).

### SVs involved with predicted gene fusions in cancer cell lines

**Fig. 4:**
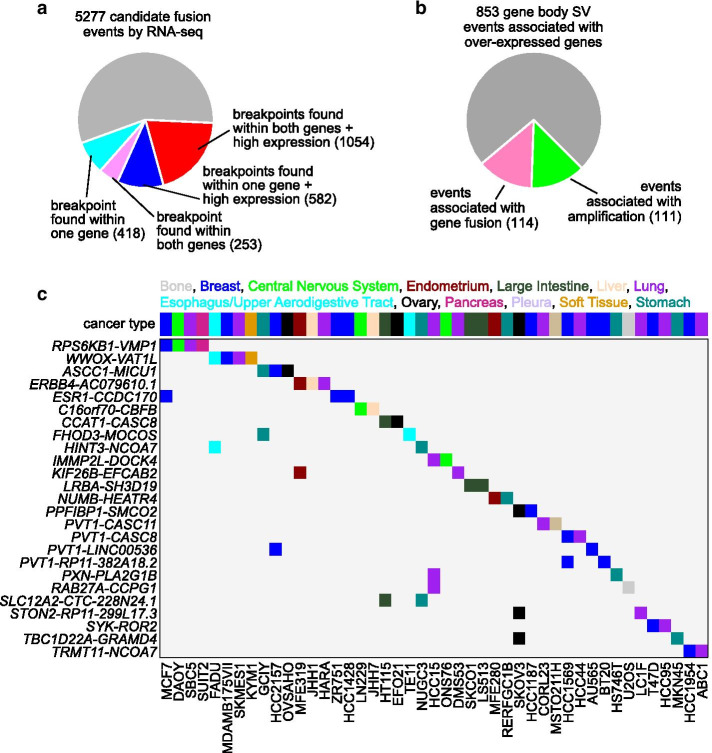
Identification of gene fusion events in cancer cell lines by both RNA-seq and WGS. **a** Out of 5277 candidate fusion events identified by RNA-seq analysis (using STAR-fusion algorithm) involving 327 cell lines with WGS data [10], numbers of events with support from somatic SV analyses. As indicated, for 2307 candidate fusion events, SV breakpoints were found within one or both genes, with and without a high expression association. A high expression association is defined here as one of the following: (1) for fusion events occurring in one or two tumors, whether for each tumor the expression of either gene was > 0.4SD from the median; or (2) whether a significant association between SV breakpoints and increased expression (*p* < 0.01, linear model incorporating tumor type and CNA) was found for either gene, either by distance metric method or by genomic region window within the gene body. **b** Of the 853 gene body SV breakpoint events associated with overexpressed genes (over-expression defined as > 0.4SD from the sample median, events involving the set of 192 genes from Fig. 2a with FDR < 10%, correcting for tumor type and CNA), the fractions of events associated with either gene fusion by combined RNA-seq and SV analysis or high-level gene amplification are indicated. **c** Gene fusions with both RNA-seq and SV support (i.e., breakpoints detected for at least one of the two genes) with high expression association (part a) and involving more than two cell lines are represented. Cancer type is indicated along the top and in the coloring of the fusion event

RNA-seq-based fusion predictions, based on chimeric sequencing reads, may be refined using somatic SV data in conjunction with SVExpress. Out of 5277 candidate fusion events identified by RNA-seq analysis [10] (STAR-fusion algorithm) involving the 327 CCLE cell lines with WGS data, 2307 (44%) corresponded to SV breakpoints found within one or both genes (Fig. 4a), and 1636 of these involved a high expression association by SVExpress. This set of 1636 fusion calls with the highest level of support involved 1604 distinct gene fusions and 226 cell lines (Additional file 1: S1), as well as the majority of within-gene SV breakpoint events involving over-expression (Fig. 4b). Twenty-five fusions were detected in more than one cell line (Fig. 4c), a number of which have previously been detected in human tumors, including *RPS6KB1*-*VMP1* [15], *WWOX*-*VAT1L* [16], *ASCC1*-*MICU1* [17], *ESR1*-*CCDC170* [18], *FHOD3*-*MOCOS* [19], *IMMP2L*-*DOCK4* [19], *LRBA*-*SH3D19* [19], *PPFIBP1*-*SMCO2* [19], *PVT1*-*CASC11* [20], *PVT1*-*CASC8* [20], *PXN*-*PLA2G1B* [21], *TBC1D22A*-*GRAMD4* [19], and *TRMT11*-*NCOA7* [19]. The well-known TMPRSS2 gene fusions in prostate cancer [22] were also detected in CCLE data by RNA-seq STAR-fusion algorithm, in VCAP and NCIH660 cell lines, but these cell lines did not have corresponding WGS data for SV calling.

### Evaluation of other public software for integrating SV with expression data

We examined our SVExpress results from the CCLE in the context of two other public software tools for integrating SV with expression data: SV-HotSpot [7] and cis-X [6]. Each of the above tools utilizes a different data integration approach. SV-HotSpot, as a first step, identifies recurrent SVs and their targeted hotspot regions and then tests which genes associated with these SV hotspots show corresponding changes in expression. Therefore, a gene that is significant by SV-HotSpot must show both an SV hotspot pattern and SV-associated altered expression. On the other hand, cis-X first identifies candidate cis-activated genes that exhibit combined allele-specific expression (ASE) and outlier high expression. These candidate cis-activated genes are then associated with any nearby SV breakpoints. The cis-X software evaluates samples individually, whereas both SV-HotSpot and SVExpress analyze all the samples in the cohort together to identify patterns of recurrence. In contrast to cis-X or SV-HotSpot, SVExpress does not rely on ASE or SV hotspot patterns, respectively. SVExpress does not assume a mechanism of deregulation for SV-associated deregulated genes, and SV breakpoints not involving TAD disruption or enhancer hijacking, for example, may still contribute to a significant gene pattern by SVExpress.

**Fig. 5:**
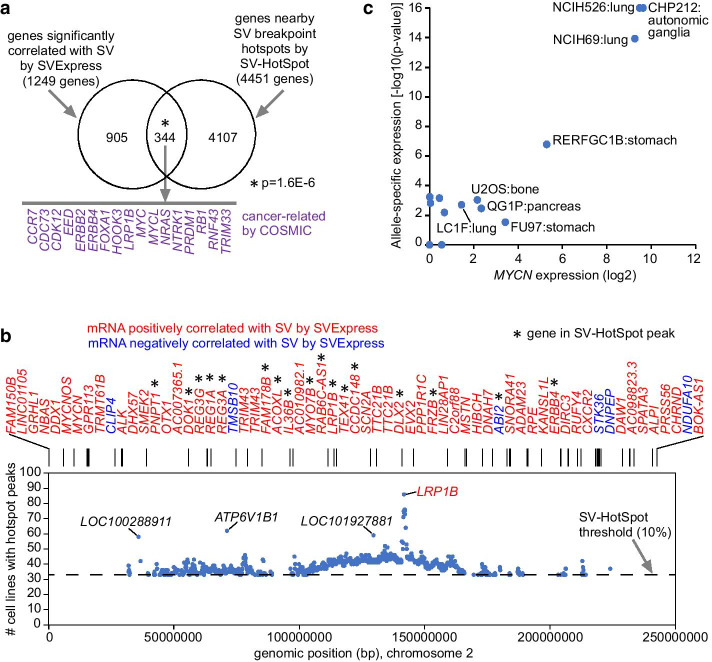
Evaluation of SVExpress results in the context of SV-HotSpot and cis-X SV analysis tools. **a** From results using the CCLE datasets, Venn diagram represents the overlap between the genes significantly correlated with SV breakpoint by SVExpress (FDR < 10%, using 1 Mb region window and correcting for cancer type and CNA) with the set of genes associated with nearby SV hotspot peaks by SV-HotSpot [7]. P-value by one-sided Fisher’s exact test. Genes listed, involving the overlap between the SVExpress and SV-HotSpot results sets, also have a previous cancer association by COSMIC [23]. **b** Survey of SV hotspot peaks across chromosome 2, by genomic position. SV-HotSpot uses a default threshold of 10% of cell lines for calling SV hotspot peaks. Along the top of the plot, the genomic positions of the genes significant by SVExpress are represented. Asterisks indicate significant SVExpress genes that also are associated with an SV-HotSpot peak. **c** For cancer cell lines with SV breakpoint within 500 kb of *MYCN*, allele-specific patterns associated with increased expression. *P* values by binomial test using cis-X [6]

We analyzed the CCLE SV dataset using SV-HotSpot. Of the 20,153 genes in the CCLE dataset that we analyzed above using SVExpress, SV-HotSpot identified 4451 genes associated with SV hotspots, defined as genomic regions with breakpoints involving more than 10% of the 327 cell lines analyzed. Of these 4451 genes, 344 overlapped with the 1249 genes significant by SVExpress (FDR < 10%, using 1 Mb genomic region window and correcting for both cancer type and CNA), this overlap being statistically significant (*p* = 1.6E−6, one-sided Fisher’s exact test, Fig. 5a). Notably, most genes significant by SVExpress would not be considered significant by SV-HotSpot, as these genes may involve fewer than 10% of cell lines and would therefore not pass SV-HotSpot’s hotspot filter. Taking chromosome 2 as an example (Fig. 5b), 17 out of 61 genes significant by SVExpress involved hotspots, including COSMIC [23] genes *LRP1B* and *ERBB4*, and COSMIC genes not involving hotspots included *MYCN* and *ALK*. The highest hotspot peak on chromosome 2 involved *LRP1B*, though other high hotspot peaks identified using SV-HotSpot did not involve any genes of interest.

An association between SV breakpoints and altered cis-regulation may be further evidenced by ASE patterns, whereby the allele with the somatic variant is the one that presumably has the aberrant expression. We used cis-X to focus here on *MYCN*, a cancer-associated gene significant by SVExpress but not SV-HotSpot. Using cis-X and the RNA-seq BAM files of the 17 cell lines with an SV breakpoint occurring within 500 kb of *MYCN*, we could observe that SV-associated up-regulation of *MYCN* appeared allele-specific (Fig. 5c). Cell lines with high *MYCN* expression tended to show ASE for *MYCN*. *MYCN*-altered cell lines included lung, autonomic ganglia, stomach, bone, and pancreas. Recently, we also used cis-X to validate ASE patterns for two genes (*TERT* and *MYB*) with SV-associated altered expression by SVExpress in a cohort of pediatric brain tumors [4]. The cis-X ASE analysis is somewhat limited by the availability of genetic markers in the region of interest.

SVExpress may be considered more user-accessible than either SV-HotSpot or cis-X, by SVExpress being compatible with Windows or macOS. Both SV-HotSpot and cis-X require BEDTools, among other dependencies, which is designed for UNIX [5]. The requirement of cis-X for RNA-seq BAM files (as part of the ASE analysis step) makes this software resource-intensive, and a high-performance computing environment is needed here. The original cis-X study featured an analysis of just 13 T-lineage acute lymphoblastic leukemias [6], but carrying out an analogous study of 327 cancer cell lines using cis-X would represent a major endeavor. In contrast, potential users of SVExpress may not be limited to highly skilled computational biologists with easy access to high-performance computing. Essentially anyone with a Windows or macOS desktop may use SVExpress. As demonstrated here, SVExpress can identify SV-associated genes of interest, which optionally can then be further examined using cis-X or SV-HotSpot, as each software represents its unique approach with associated strengths.

## Discussion

The SVExpress tools presented here enable general users to carry out integrative analyses of somatic SVs and gene expression data in cancer samples. As recent efforts by the Pan-Cancer Analysis of Whole Genomes (PCAWG) consortium and others have demonstrated [24], combined whole-genome DNA and RNA sequencing of cancers is becoming a standard component of cancer genomics studies. As compared to results from pan-cancer analyses, individual cancer types may show a different set of genes with altered expression in association with somatic SV breakpoints [2]. Therefore, future studies may use SVExpress to explore individual cancer types, as greater numbers of patient samples with profiling data are available.

Through analysis of cell line data, we found here that the overall phenomenon of somatic SV-mediated cis-regulatory alterations, as previously observed in human tumors of various types [1–4], is also at work in cell lines, though with a somewhat different set of altered genes. Aspects of this phenomenon, as observed now in both cell lines and human tumors, include the following: hundreds of genes recurrently impacted, SV breakpoints as far as 1 Mb from the gene contributing to deregulation, rearrangements involving widespread CNA patterns, many more genes with increased over decreased expression associated with SV breakpoints, and over-expressed and under-expressed genes respectively representing known oncogenes and tumor suppressor genes. As intended, our analytical approach does not assume the specific mechanism of altered expression, as there may be multiple mechanisms involved for any given gene across multiple samples. SVExpress may reveal correlations, though correlation does not necessarily demonstrate causation or point to a specific mechanism. Still, the ability of SVExpress to identify translocated enhancers involving some altered expression events may provide clues as to the mechanism of altered cis-regulation. However, such enhancer associations might warrant experimental confirmation.

## Conclusion

Our SVExpress tools allow computational biologists with a working knowledge of R to identify SV events that may involve gene fusions (e.g., a breakpoint within a given gene associated with its over-expression), gene disruption (breakpoint within a gene associated with loss of expression), enhancer hijacking, or TAD disruption. SVExpress is freely available for academic or commercial use at https://github.com/chadcreighton/SVExpress. Provided with the SVExpress macros and R-code are example data from the Cancer Cell Line Encyclopedia [10], along with instructions for use. All source code (R and Visual Basic for Applications) is available.

### Availability and requirements

Project name: SVExpress.

Project home page: https://github.com/chadcreighton/SVExpress.

Operating systems: Windows or macOS.

Programming languages: R and Excel Visual Basic for Applications (VBA).

Other requirements: none.

License: open-source.

Any restrictions to use by non-academics: none.

## Supplementary Information

**Additional file 1.** Data File S1: SV-expression integrative analysis results from the Cancer Cell Line Encyclopedia (CCLE). Provided are the complete set of gene-level correlations between expression and nearby somatic SV breakpoint in the 327 CCLE cell lines with WGS data, according to region examined (e.g., 0–100 kb upstream, 0–100 kb downstream, within the gene body, or 1 Mb upstream or downstream) and the regression model applied (with or without CNA correction). For the 1 Mb region window, relative gene distances of the breakpoints are weighted in the model. A separate tab provides the top genes for any region with FDR < 10% (correcting for CNA, with FDR by Story and Tibshirani method). Another tab provides the SVs associated with enhancer hijacking and gene over-expression. Another tab provides information on gene fusion predictions made by RNA-seq that also have support from WGS analysis of the 327 cell lines. Provided as an Excel file. (XLSX 9301 KB)

## Data Availability

SVExpress is freely available for academic or commercial use at https://github.com/chadcreighton/SVExpress. The CCLE datasets are available at https://portals.broadinstitute.org/ccle/data.

## Abbreviations

CCLE: Cancer Cell Line Encyclopedia
SV: Structural variant
CNA: Copy number alteration
WGS: Whole-genome sequencing
TCGA: The Cancer Genome Atlas
PCAWG: Pan-cancer analysis of whole genomes
